# Evaluation of Safety and Efficacy of an Inactivated Marker Vaccine against *Bovine alphaherpesvirus 1* (BoHV-1) in Water Buffalo (*Bubalus bubalis*)

**DOI:** 10.3390/vaccines9040355

**Published:** 2021-04-07

**Authors:** Stefano Petrini, Alessandra Martucciello, Francesco Grandoni, Giovanna De Matteis, Giovanna Cappelli, Monica Giammarioli, Eleonora Scoccia, Carlo Grassi, Cecilia Righi, Giovanna Fusco, Giorgio Galiero, Michela Pela, Gian Mario De Mia, Esterina De Carlo

**Affiliations:** 1 National Reference Centre for Infectious Bovine Rhinotracheitis (IBR), Istituto Zooprofilattico Sperimentale Umbria-Marche, “Togo Rosati,” 06126 Perugia, Italy; m.giammarioli@izsum.it (M.G.); e.scoccia@izsum.it (E.S.); c.righi@izsum.it (C.R.); m.pela@izsum.it (M.P.); gm.demia@izsum.it (G.M.D.M.); 2 National Reference Centre for Hygiene and Technology of Breeding and Buffalo Production, Istituto Zooprofilattico Sperimentale del Mezzogiorno, 84131 Salerno, Italy; alessandra.martucciello@cert.izsmportici.it (A.M.); giovanna.cappelli@izsmportici.it (G.C.); carlo.grassi@izsmportici.it (C.G.); giovanna.fusco@cert.izsmportici.it (G.F.); giorgio.galiero@cert.izsmportici.it (G.G.); esterina.decarlo@cert.izsmportici.it (E.D.C.); 3 Research Centre for Animal Production and Aquaculture, Monterotondo, 00015 Rome, Italy; francesco.grandoni@crea.gov.it (F.G.); giovanna.dematteis@crea.gov.it (G.D.M.)

**Keywords:** BoHV-1, marker vaccines, water buffalo

## Abstract

Recent studies have explored the seropositivity of *Bovine alphaherpesvirus 1* (BoHV-1) in water buffaloes, suggesting the urgency for developing strategies to eradicate the virus involving both cattle and water buffaloes. However, in Europe, the glycoprotein E (gE) deleted marker vaccines against BoHV-1 are commercially available only for the cattle industry. This study, for the first time, evaluated the safety and efficacy of a commercial inactivated gE-deleted marker vaccine in water buffalo. Five animals devoid of BoHV-1-neutralizing antibodies were vaccinated via intramuscular route. Five additional animals served as an unvaccinated control group. Sixty days after the first immunization, all animals were experimentally infected with a virulent BoHV-1via intranasal route. A detectable BoHV-1-humoral immune response was observed in the vaccinated group on post-vaccination day 30, whereas the antibodies appeared on post-challenge day 10 in the control group. Moreover, the vaccinated animals neither show viral shedding nor clinical signs compared to the control upon challenge. However, post-challenge, the BoHV-1-specific humoral and cell-mediated immune responses were significantly more increased in vaccinated animals than the control animals. Overall, the present study provides evidence of both the safety and efficacy of an inactivated gE-deleted marker vaccine against BoHV-1 in water buffaloes.

## 1. Introduction

Water buffaloes originated in Asia, but are currently found on all five continents. To date, in the world, there are about 210 million buffaloes [1] of which approximately 4.13 million are bred in Italy and are located mainly in Central and South Italy (Lazio and Campania Regions), to produce traditional dairy products (Ministry of Health, National Database as of 31 December 2020).

Herpesviruses, the members of the *Herpesviridae* family, are known to infect and cause diseases in animals and humans. To date, more than 200 etiologic agents have been reported in the *Herpesviridae* family, of which *Bovine alphaherpesvirus 1* (BoHV-1) and *Bubaline alphaherpesvirus 1* (BuHV-1) belonging to the subfamily, *Alphaherpesvirinae* and genus *Varicellovirus,* have been reported to infect water buffalo (*Bubalus bubalis*) [2].

The BoHV-1 infection that causes severe losses to the cattle industry worldwide is associated with two different clinical syndromes, namely infectious bovine rhinotracheitis (IBR) and infectious pustular vulvovaginitis (IPV). In addition, it is also associated with a variety of clinical signs, including fever, dyspnea, conjunctivitis, nasal discharge, vaginitis, balanoposthitis, abortions, enteritis, and encephalitis [3,4]. In contrast, in water buffalo adults, although the virological and serological positivity of BoHV-1 has been demonstrated, the clinical signs of disease have not been reported [5]. However, in water buffalo calves, BuHV-1, closely related to BoHV-1, has been shown to be associated with fever, cough, sneezing, wheezing, nasal and ocular secretions, loss of appetite, depression, and lethargy, while no clinical signs were observed in adult water buffalo [6]. Moreover, BuHV-1 has also been shown to be associated with abortion [7,8].

BoHV-1 has experimentally been shown to infect other animals than its primary host, including goat, sheep, red deer, and reindeer [5,9], and its seropositivity has also been documented in water buffalo [10]. However, in the past, the control of BoHV-1 in water buffalo was discouraged because an ELISA test was not available. Moreover, the virus neutralization test between BoHV-1 and BuHV-1 was not able to differentiate these two viruses because of their high genomic homology that shows a sequence nucleotide identity of approximately 87.7% for glycoprotein B (gB) [9] and 77.0% for glycoprotein E (gE) [11].

Recently, a new indirect ELISA test based on BuHV-1 and BoHV-1 gE has been developed to differentiate these viruses based on their specific infection status [11,12]. Moreover, Scicluna et al. [13] have described the circulation of both infections in water buffalo observed in several herds, suggesting the concomitance of the infection by both viruses in buffaloes and cattle [13]. In Italy, water buffalo and cattle are often bred together, thus representing a risk factor for cross infections. Therefore, it has been suggested that the systematic IBR eradication plan should involve both cattle and water buffaloes [14]. The protective measures aimed at controlling BoHV-1 infection in cattle include the administration of live attenuated or inactivated vaccines. In addition, marker vaccines that lack one or more genes responsible for glycoprotein or enzyme synthesis have also been used [15,16,17,18]. The strategy of marker vaccination that differentiates the vaccinated animals is called Differentiating Infected from Vaccinated Animals (DIVA) [18]. The deletion of the gene encoding glycoprotein E (gE) of BoHV-1 is the most commonly used genetic marker for the BoHV-1 DIVA vaccine. Vaccination with this type of marker vaccine makes it possible to differentiate the immunized animals (gE-negative) from those infected with wild type BoHV-1 or vaccinated with traditional non-deleted vaccines (gE-positive) by gE-specific ELISA test. *Bos taurus* and *Bubalus bubalis* have a common ancestor and share a high genomic identity (>91%), with approximately 3% divergence between buffalo and cattle genes; this may have practical implications, for example the possibility of cross-species application of vaccine development. Therefore, we hypothesized that the gE-deleted marker vaccine originally registered for cattle could be effective against the BoHV-1 in water buffalo, a potential carrier of the virus. To test this hypothesis, the present study was aimed to evaluate the safety and efficacy of a vaccination protocol in water buffalo against BoHV-1 using an inactivated gE-deleted marker vaccine. The findings revealing the safety and efficacy of the gE-deleted marker vaccine originally registered for cattle could be useful for developing safe and effective IBR eradication strategies.

## 2. Materials and Methods

### 2.1. Virus

The wild-type strain 16453/07 TN of BoHV-1 was selected for this study. The strain was used at the fifth passage on Madin-Darby Bovine Kidney (MDBK) cell cultures at a titer of 10^6.74^ median tissue culture infectious dose (TCID_50_)/mL. This virus was isolated during an IBR outbreak that occurred in 2007 in a dairy herd located in central Italy (Petrini, unpublished data).

### 2.2. Vaccine

A commercial inactivated gE-deleted marker vaccine (Bovilis ^®^ IBR marker inactivatum, Intervet International B.V., Boxmeer, Holland) was used in this study. Two doses of the vaccine at 2 mL were administered to each animal at an interval of 30 days starting at the age of 15 months. The vaccine was injected intramuscularly (i.m.) into the neck muscle.

### 2.3. Experimental Design

Ten water buffaloes devoid of BoHV-1 neutralizing antibodies were used. All the animals in this study were from a single water buffalo breeding center located in the south of Italy (Campania region). According to the farm records, no vaccine against BoHV-1 had been used before, and no recent history of respiratory disease was registered. The animals were housed in an experimental farm and fed twice per day with a unified mixture and water ad libitum. According to the European legislation on the protection of animals used for scientific purposes, maintenance and experimental protocols were established [19]. Furthermore, the Italian Ministry of Health approved the experiments under authorization number 859/2017-PR.

The number of animals in each group was determined through the sampling procedure envisaged for an experimental clinical study with an error of 1% and a study power of 80%. For the proportion of the appearance of the event (event = antibody responses), the percentages of 0% and 90% were considered in the control and the experimental group, respectively.

The buffaloes were divided into two groups of five animals each. The animals in the first group (A) were immunized with a commercial inactivated gE-deleted marker vaccine. The second group (B) served as an unvaccinated control group. The animals in each group were housed in separate pens.

Sixty days following the first immunization, all animals were subjected to challenge infection with a wild-type BoHV-1 strain. Each water buffalo received 5 mL × 10^6.74^ TCID_50_/mL administered via the intranasal route.

The buffaloes were kept under observation for 59 days after the challenge, and rectal temperatures were taken daily. Fever was confirmed when the rectal temperature was greater than 38.2 °C [5]. Any appearance of adverse reactions after vaccination was constantly monitored by veterinary supervision.

On the day of the first vaccination (time 0), at 30 and 60 post-vaccination days (PVDs), serum samples were collected from each water buffalo and tested for the presence of BoHV-1 antibodies. Whole blood and serum samples were collected from all animals at 0, 2, 4, 7, 10, 15, 30, and 59 post-challenge days (PCDs) and tested for the presence of BoHV-1 antibodies and to assess flow cytometry analysis. Simultaneously, serum samples were collected from all animals to test for BoHV-1/BuHV-1 discrimination by ELISA test.

Nasal swabs in transport fluid Minimum Essential Medium (MEM) were obtained from each buffalo at 0, 2, 4, 7, 10, 15, 30, and 59 PCDs and used for virus isolation and titration assays.

### 2.4. Virus Isolation

Serial dilutions ranging from 10^–1^–10^–9^ of the supernatants from each nasal swabbing were inoculated at a volume of 0.1 mL into three wells of a 24-well plastic plate containing monolayers of MDBK cell cultures grown in MEM. The cells were provided by Biobanking of Veterinary Resources (BVR), Brescia, Italy and identified with the code BS CL 63. After 60-min incubation at 37 °C in a 5% CO_2_ atmosphere, 1 mL of MEM enriched with 2% FCS (BioWhittaker Inc., Walkersville, MD, USA) was added to each well. The positive control was prepared from MDBK cell cultures infected with Los Angeles reference strain 01/17 of BoHV-1. MDBK cell cultures free of BoHV-1 were used as a negative control. The plates were incubated for 7 days at 37 °C in a 5% CO_2_ atmosphere and observed daily for the appearance of cytopathic effect (CPE). Virus titer was determined according to Reed and Muench [20] and expressed as TCID_50_/mL. The virus recovered from the samples was identified as BoHV-1 by direct immunofluorescence assay using an anti-BoHV-1 monoclonal antibody labeled with fluorescent isothiocyanate (Bio 026, Bio-X Diagnostic S.A., Rochefort, Belgium).

### 2.5. Blood Sample Collection

Whole blood samples (approximately 7 mL) were collected from the jugular vein into K3-EDTA, Li-Heparin, and anticoagulant-free vacutainer tubes (Vacuette^®^, Greiner Bio-One Italy, Rome) for hematological, flow cytometry, and serological analyses, respectively.

The serum samples were centrifuged at 850× *g* for 30 min at 4 °C to extract the serum. The samples were transported to the laboratory within 2 h of collection before testing. Afterward, all samples were stored at −20 °C for further serological studies. A detailed description of sample processing for hematological and flow cytometry analysis is described in Section 2.8.

### 2.6. Neutralization Test

The serum samples were tested using the protocol described by the OIE Manual of Diagnostic Tests and Vaccines for Terrestrial Animals [21]. Briefly, 50 µL of undiluted serum samples and two-fold dilutions of each were mixed with 50 µL of 100 TCID_50_ of BoHV-1 (Los Angeles reference strain 01/17) into three wells of 96-well microtiter plates. The serum samples were incubated at 37 °C for 24 h, and then 30,000 MDBK cells in 100 µL were added to each well. After 4 days of incubation at 37 °C, the plates were read using an inverted tissue culture microscope to determine the presence of CPE. Neutralization titers were expressed as the highest dilution inhibiting cytopathology.

### 2.7. ELISA Tests

Three commercial ELISA tests (IDEXX IBR gE Ab test, Maine, USA; IDEXX IBR gB X3 Ab, Maine, USA; In 3Diagnostic, Eradikit^TM^ BoHV1-BuHV-1 Discrimination Kit) were used in parallel to examine the collected sera. The protocols described by the kit manufacturer were followed, and the results were also expressed according to the manufacturer’s instructions. The microplates were read using an automated plate reader, and the data were analyzed using Magellan software (Tecan AG, Männedorf, Switzerland).

### 2.8. Hematological and Flow Cytometry Analysis

Total and differential leukocyte counts were performed using a hematology analyzer Cell-Dyn 3700 SL (Abbott, Abbott Park, IL, USA), according to the standard operating procedure. A pre-trial study was performed to evaluate the cross-reactivity of anti-human CD79a-clone HM47 (BD Pharmigen, Becton Dickinson, Plymouth, UK) and anti-human CD21-clone LT21 (Thermo Fisher Scientific, Waltham, MA, USA) to identify water buffalo B lymphocytes and the CD21^+^ subset, respectively. A similar expression pattern to the target species (human and cross-reactive bovine), percentage of positive cells comparable with previously validated clones, or absence or reduced background, allowed the validation of these clones as previously described [22].

To evaluate the T lymphocyte population and the relative subsets, a four-color panel was used: FITC anti-CD8 (clone CC63), Zenon^®^ PE anti-CD4 (IL-A11a), LYNX^®^ PE-Cy7 anti-δ chain (clone GB21a), and LYNX^®^ APC anti-CD3 (clone MM1a). The in-house labeling methods were performed using Zenon^®^ (Thermo Fisher Scientific, Waltham, MA, USA) and LYNX^®^ (Bio-Rad Laboratories, Hercules, CA, USA) following the manufacturer′s instructions. Fifty microliters of whole blood were incubated with saturating concentrations of monoclonal antibodies in a final volume of 100 µL with phosphate-buffered saline (PBS, pH 7.2) for 15 min at 4 °C in the dark. Erythrocyte lysis was performed by adding 1.0 mL of Tris-buffered ammonium chloride solution (0.87% *w*/*v*, pH 7.3) for 10 min. After the addition of 2.0 mL cold PBS, samples were centrifuged at 300× *g* for 5 min at 4 °C and resuspended in 150 µL cold PBS.

A two-color flow cytofluorimetric panel: PE-anti-CD21 and APC anti-CD79a, was performed to identify B lymphocytes, using the PerFix nc Kit (Beckman Coulter, Brea, CA, USA). Briefly, 50 μL of whole blood placed in a 5 mL PP capped test tube (Sarsted, Nümbrecht, Germany) were incubated with 5 µL of the fixative reagent for 15 min at 22 °C. Then, 300 µL permeabilizing reagent was added and immediately incubated with PE anti-CD21 and APC anti-CD79a monoclonal antibodies for 30 min in the dark at RT. Finally, 2 mL of the final 1 x reagent solution was added to each tube, and the cells were stored at RT until flow cytometric acquisition.

All labeled samples were immediately acquired using a CytoFLEX flow cytometer, and the data were analyzed using Kaluza software v. 2.1 (Beckman Coulter, Brea, CA, USA).

### 2.9. Statistical Analyses

For all distributions, the normality of the data was verified using the Shapiro–Wilk test; therefore, non-parametric tests were used when normality was not verified, and parametric tests were used for normally distributed data. The titers of antibodies were measured on a logarithmic scale with base 10. The means of the titers were calculated for each animal group and for all sampling times. The Wilcoxon Mann–Whitney test was used to verify serological differences between the control and experimental groups. For the evaluation of the hematological data and those of the flow cytometry, two tests were used based on the distribution of the data: Student’s *t*-test and the Kruskal–Wallis test. All statistical analyses were performed using Stata software v.11.2 (StataCorp LCC, Texas, TX, USA) at a significance level of *p* ≤ 0.05.

## 3. Results

### 3.1. Clinical Response

The tested vaccine did not induce any clinical signs or adverse reactions in the immunized water buffaloes within the 60-day post-vaccination period. The rectal temperatures were within normal values and were similar to the control values.

After challenging on 60 PVD, no clinical signs were observed in any of the immunized water buffaloes. On the contrary, in unvaccinated controls, on PCD 7, three animals showed nasal mucus discharge, lesions at the nasal mucosa consisting of pseudomembranes associated with mucopurulent exudate, and dyspnoea and cough. In addition, the rectal temperatures were slightly increased up to 38.7 °C from PCD 2 to PCD 5 in control animals.

### 3.2. Virus Shedding

After challenge infection, the vaccinated animals did not shed wild-type BoHV-1, whereas the control animals shed the virus on PCD 2, 4, and 7. The mean titer of the virus recovered from the unvaccinated controls on PCD 2 was 10^6.24^ TCID_50_/mL. From PCD 2 to PCD 7, the titers were dropped by 1.74 log units, and the number of unvaccinated animals that shed the virus decreased from 5 to 1. None of the animals in the control group shed the virus on or after PCD 10 (Table 1).

### 3.3. Serological Investigations

A progressive increase in the BoHV-1 neutralizing antibody titer was detected in the vaccinated animals that presented a mean titer of 1.02 log_10_ (*p* = 0.0052) and 1.75 log_10_ (*p* = 0.0052) on PVD 30 and 60, respectively. In the control group, no neutralizing antibodies were detected. At PVD 30 and PVD 60, the vaccinated animals were seropositive for gB-ELISA and negative for gE-ELISA and BoHV1-BuHV1 discrimination Kit. Likewise, no seroconversion was detected in the unvaccinated controls (Table 2).

The neutralizing antibody titer of vaccinated animals increased after challenge infection, reaching a value of 2.95 log_10_ (*p* = 0.0088) on PCD 15 and 3.07 log_10_ (*p* = 0.0086) on PCD 30. The same titer persisted until PCD 59 (*p* = 0.0084). In the control group, NAs were detected on PCD 15 with a mean titer of 1.32 log_10_. This titer increased by 0.43 log_10_ on PCD 59. Conversely, in vaccinated animals, a positive signal for gE was detected only on PCD 59. In addition, no seroconversion was detected for the BoHV1-BuHV1 discrimination kit throughout the entire experimental period. In contrast, in the animals of the control group, antibodies for gB were detected on PCD 10, and the ensuing BoHV-1 seropositivity was succeeded until the end of the experiment. In the same group, the seropositivity for gE and the BoHV1-BuHV1 discrimination kit was detected on PCD 15 and sustained until the end of the experiments (Table 3).

### 3.4. Flow Cytometry

The measurements of B and T lymphocyte subsets showed significant differences between vaccinated buffaloes and unvaccinated controls during the entire observation period (Table 4) and at each experimental time point (Table 5). Interestingly, the vaccinated animals showed higher percentages of γδ and αβ CD4^+^ T lymphocytes, CD21^+^ B lymphocytes, and CD4^+^/CD8^+^ ratio than unvaccinated controls. However, the animals in group A showed higher percentages of αβ CD8^+^ T lymphocytes than group A throughout the experiment.

## 4. Discussion

The EU’s “Animal Health Law” [23] and the subsequent Regulations [24,25] include IBR among the diseases subject to control or eradication plans in the following species: *Bison* ssp., *Bos* ssp., and *Bubalus* ssp. In cattle, the DIVA strategy is considered the first line of intervention for eradication programs in areas where IBR infection has a high prevalence. This strategy enables the differentiation of animals immunized with gE-deleted marker vaccines (gE-negative) from those infected with either the wild-type virus or immunized with traditional non-deleted marker vaccines (gE-positive) through diagnostic tests specific for gE of BoHV-1 [17,18,26,27].

Though it is known that BoHV-1 can cross the species barrier and infect other animals, including water buffaloes, at present, little information is available on the epidemiological role of *Bubalus spp.* on BoHV-1 infection [8,10,14,28,29,30]. Furthermore, in Italy, in a study conducted on 1756 serum samples collected from buffaloes in central Italy (Lazio Region), 30.6% of samples were seropositive to BoHV-1, whereas 42.0% samples were seropositive to BuHV-1 [13]. In contrast, in another study conducted on 1089 serum samples (Piedmont and Campania Regions), 59% of the samples reacted positively to ELISA test irrespective to BoHV-1 or BuHV-1 antigen, and 86.4% were reactive to BuHV-1 only, whereas 11.8% were positive for both antigens and were classified as inconclusive. This study reported a low percentage of sera reactive to BoHV-1 (1.8%) and suggested that BuHV-1 could be the main circulating alphaherpesvirus infection in Mediterranean water buffaloes [12]. It is known that inactivated gE-deleted marker vaccines induce a high immune response in cattle [17,27], while no information is available regarding the induction of the immune response by these vaccines in the *Bubalus bubalis* against BoHV-1.

This study, for the first time, demonstrates the safety and efficacy of an inactivated gE-deleted marker vaccine (Bovilis^®^ IBR marker inactivatum, Intervet International B.V., Boxmeer, Holland) authorized in the European Union for cattle, in *Bubalus bubalis*.

We performed several experiments using an inactivated gE-deleted marker vaccine administered via the i.m. route. The results demonstrated that the product did not induce any clinical signs or adverse reactions. The results of this study, in agreement with those published previously on cattle [17,31], suggested that in water buffalo, there is no risk of adverse reactions following the administration of the inactivated gE-deleted marker vaccine. Moreover, the outcomes of the present study demonstrate no clinical signs after immunization with the modified live gE-deleted marker vaccine. The results are in accordance with Montagnaro et al. [32].

After the experimental infection in the vaccinated animals, the rectal temperature was within the physiological range, and the vaccinated animals did not show any clinical signs compared to the controls. The control group showed clinical signs and a slight increase in temperature. Nasal swabs showed an absence of shedding in the vaccinated group compared to the unvaccinated group (*p* = 0.3173) during the entire experimental period. In the control group, the virus was isolated up to PCD 7. In our study, the values of rectal temperatures obtained are similar to those published by other studies [5], while the clinical results obtained in the control group differ from those of Scicluna et al. [5], and the same results are similar to Montagnaro et al. [32]. As for virus excretion, the results of our study differ from those obtained from other studies, where BoHV-1 was isolated from nasal swabs at a low titer (10^−1^ dilution) from PCD 7 to PCD 14 [5]. In water buffaloes immunized with an attenuated gE-deleted marker vaccine against BoHV-1 and after experimental infection with BuHV-1, no clinical signs referable to BoHV-1 were observed throughout the experimental period, and a significant reduction in virus shedding (BuHV-1) was observed up to PCD 10 [32]. In addition, different studies reporting an inactivated vaccine against BoHV-1 administered to cattle via the i.m. route did not show fever, whereas nasal discharge and dyspnea were observed in one calf only. In addition, the virus was shedding up to PCD 10 [15].

The humoral immune response to vaccination was evident on PVD 30, and an increase in antibodies on PVD 60 compared to the control group was observed. Similar results were obtained in a study conducted by Montagnaro et al. using a modified live gE-deleted marker vaccine in water buffalo [32]. Moreover, these results agree with the results of previous studies on cattle immunized with two inactivated gE-deleted marker vaccines [17]. In contrast, other studies described a low induction of NA against BoHV-1 after immunization with inactivated gE-deleted marker vaccines [33]. Furthermore, in buffalo calves, an increase in NA was also observed in another study using different inactivated vaccines containing foot-and-mouth disease (FMD), bovine ephemeral fever (BEF) and *Pasteurella multocida* [34,35,36].

The vaccinated animals showed negative results for the gE-ELISA and BoHV1-BuHV1 discrimination kit during the vaccination period. The results obtained from the gE-ELISA and BoHV1-BuHV-1 discrimination kit, excluding that during the vaccination period, showed that both BoHV-1 and BuHV-1 field viruses could have circulated within the experimental group. Indeed, water buffaloes naturally or experimentally exposed to BoHV-1 or BuHV-1 are shown to be positive for gE-ELISA or BoHV1-BuHV1 ELISA antibodies [6,11].

In this study, we detected gE-ELISA positivity in vaccinated animals on PCD 59, whereas seroconversion to gE-ELISAs on PCD 15 was observed in the controls. The results obtained from the vaccinated group differ from those obtained using a gE-deleted marker vaccine in cattle, where several reports have shown that seroconversion to gE protein occurs from 2–4 weeks after the experimental infection [33,37,38].

Furthermore, the NA results showed an increase in the antibody titer (*p* = 0.0084) in the vaccinated animals compared to the controls until the end of the experiment. In addition, in vaccinated animals, a positive result was observed for gB-ELISA during the experimental period. In the controls, gB-positive results were evidenced on PCD 10. These results are similar to those published in cattle by other studies and demonstrate that animals immunized with inactivated gE-deleted marker vaccines constantly increase their humoral immune response and are protected against experimental infection, while the controls seroconvert to gB-ELISA and NA after PCD 10–15 [15,39,40].

During the experimental period, the vaccinated animals were negative for the BoHV1-BuHV1 discrimination kit, while the control animals were positive for BoHV-1 on PCD 15. Given that the BoHV1-BuHV1 discrimination kit is based on a single epitope located in the gE/gI complex, the monoclonal antibody used in this test might have failed to recognize the previously mentioned epitope due to a high titer of direct antibodies against other BoHV-1 glycoproteins located near the gE/gI complex (e.g., 3.07 log_10_ of NA), which might have inhibited binding to monoclonal antibodies due to steric interference. This hypothesis is supported by the results obtained by Nogarol et al. [11].

Flow cytometric analysis showed that the highest total lymphocyte count, obtained by hematological test, in the vaccinated group (5030 vs. 4100 cells/µL) was due to the significant increase in total B lymphocytes CD21^+^ subset, and γδ and αβ CD4^+^ T lymphocyte subsets (Table 4). Furthermore, these differences were confirmed at each time point, and several parameters showed statistically significant differences around PCD 7 or PCD 10 (Table 5). These results highlight how these time points represent a turning point (critical points) in the immune response [22]. Studies in cattle have shown that the peak activity of cell-mediated immune responses occurs at 7–10 days post-infection and correlates with recovery from infection and before significant antibody is detectable [41]. Although the two groups showed the same trends in the immune response, the non-statistically significant differences observed at some time points could be explained by the different speeds and intensities of the immune response within the two groups, depending on individual response (data not shown). Our results demonstrate that multicolor flow cytometry can be a valuable support for assessing immune response during the execution of the water buffalo immunization protocol.

This study was conducted under experimental conditions in a rather low number of animals, for obvious practical reasons. Nevertheless, we can speculate that our findings are valuable even in a more realistic context, due to low genetic divergence and standardized living conditions of the two domesticated species. However, further studies are required to validate the efficacies of the vaccine in water buffalo farms under field conditions. Indeed, it is known that the immune response induced by the vaccine under field conditions can vary based on different parameters, including the geographical position of the farm, weather, nutrition, and general health of the herd. Therefore, for successful inclusion of the vaccine for systemic eradication of IBR, subsequent studies should be carried out to evaluate (i) other IBR deleted marker vaccines following different BoHV-1-BuHV-1 experimental infections; (ii) the duration of passive immunity in water buffalo calves following vaccination in pregnant water buffaloes with gE-deleted marker vaccines; and (iii) the protection of water buffalo calves by passive immunity against experimental infection using virulent BoHV-1 virus.

## 5. Conclusions

In conclusion, the results of this study indicate that vaccination of *Bubalus bubalis* with an inactivated gE-deleted marker vaccine authorized for cattle was able to protect water buffaloes against BoHV-1 experimental infection, as shown by any clinical signs and virus shedding. These results also demonstrate that an inactivated gE-deleted marker vaccine could be useful owing to its safety and effectiveness for Buffalo species. Collectively, these findings suggest that this type of vaccination can be used in water buffalo under the new European Regulations of the “Animal Health Law”, which can control IBR in *Bubalus bubalis.*

## Figures and Tables

**Table 1:** BoHV-1 isolation from water buffaloes immunized against BoHV-1 using an inactivated gE-deleted marker vaccine and challenge infected with virulent BoHV-1.

Group	Virus Isolation and Titration after Challenge Infection on the Day ^a^							
	0	2	4	7	10	15	30	59
A	-	N.I.	N.I.	N.I.	N.I.	N.I.	N.I.	N.I.
B	-	6.24(5) ^b^	5.18(4)	4.50(1)	N.I.	N.I.	N.I.	N.I.

A, Vaccinated group; B, Control group; ^a^ Reciprocal value of the negative log of the TCID_50_/mL (group mean value); ^b^ The number of water buffaloes from which the virus was isolated are shown in brackets; N.I., Not isolated.

**Table 2:** Antibody response of water buffaloes immunized against BoHV-1 using an inactivated gE-deleted marker vaccine.

Group		Post-Vaccination Day (PVD)		
		0	30	60 ^f^
A	gE-ELISA ^a^	-	-	-
	gB-ELISA ^b^	-	+	+
	ELISA ^c^	-	-	-
	NA ^d,e^	<1.00	1.02	1.75
B	gE-ELISA ^a^	-	-	-
	gB-ELISA ^b^	-	-	-
	ELISA ^c^	-	-	-
	NA ^d,e^	<1.00	<1.00	<1.00
	*p*-Value	-	0.0052	0.0052

A, Vaccinated group; B, Control group; ^a^ IDEXX IBR gE Ab test, Maine, USA; ^b^ IDEXX IBR gB X3 Ab, Maine, USA; ^c^ IN3Diagnostic Eradikit™ BoHV1-BuHV1 discrimination kit, Turin, Italy; ^d^ NA, neutralizing antibody; ^e^ Expressed as log_10_ of the reciprocal of the highest dilution inhibiting cytopathogenic effects (mean value); ^f^ The day of the challenge; *p*-value indicating the significant differences in NA titer between vaccinated buffaloes and unvaccinated controls.

**Table 3:** Antibody response of water buffaloes immunized against BoHV-1 using an inactivated gE-deleted marker vaccine and challenge infected with virulent BoHV-1.

Group		Post-Challenge Day (PCD)							
		0	2	4	7	10	15	30	59
A	gE-ELISA ^a^	-	-	-	-	-	-	-	+
	gB-ELISA ^b^	+	+	+	+	+	+	+	+
	ELISA ^c^	-	-	-	-	-	-	-	-
	NA ^d,e^	1.75	1.81	1.87	2.11	2.53	2.95	3.07	3.07
B	gE-ELISA ^a^	-	-	-	-	-	+	+	+
	gB-ELISA ^b^	-	-	-	-	+	+	+	+
	ELISA ^c^	-	-	-	-	-	+ ^	+ ^	+ ^
	NA ^d,e^	<1.00	<1.00	<1.00	<1.00	<1.00	1.32	1.63	1.75
	*p*-value	0.0052	0.0047	0.0052	0.0052	0.0052	0.0088	0.0086	0.0084

A, Vaccinated group; B, Control group; ^a^ IDEXX IBR gE Ab test, Maine, USA; ^b^ IDEXX IBR gB X3 Ab, Maine, USA; ^c^ IN3Diagnostic Eradikit™ BoHV1-BuHV1 discrimination kit, Turin, Italy; ^d^ NA, neutralizing antibody; ^e^ Expressed as log_10_ of the reciprocal of the highest dilution inhibiting cytopathogenic effects (mean value); + ^, positive to BoHV-1; *p*-value indicating the significant differences in NA titer between vaccinated buffaloes and unvaccinated controls.

**Table 4:** Comparison of B and T lymphocytes subset values between buffaloes immunized against BoHV-1 using an inactivated gE-deleted marker vaccine (Group A) and unvaccinated control buffaloes (Group B) after challenge infected with virulent BoHV-1.

Item	Group A		Group B		*p*-Value *^,^**
	Mean	SE ^f^	Mean	SE ^f^	
Lymphocytes (%) ^a^	48.51	2.21	49.15	0.97	0.7776 *
Lymphocytes (cells/µL) ^a^	5030	294	4100	216	0.0068 *
B Lymphocytes (%) ^b^	27.36	1.13	24.81	0.70	0.0588 **
B Lymphocytes (cells/µL) ^c^	1360	104	1012	59	0.0024 *
B Lymphocytes CD21^+^ (%) ^b^	84.43	0.95	76.40	0.89	0.0000 *
B Lymphocytes CD21^+^ (cells/µL) ^c^	1149	90	763	39	0.0001 *
γδT lymphocytes (%) ^d^	58.71	1.33	48.42	1.60	0.0000 **
αβ T CD4^+^ (%) ^e^	73.04	0.62	68.56	0.16	0.0000 **
αβ T CD8^+^ (%) ^e^	16.04	0.56	21.62	0.70	0.0000 **
αβ CD4^+^/ αβ CD8^+^^e^	4.84	0.22	3.34	0.14	0.0000 **

^a^ Percentages and absolute values obtained by Cell Dyn 3700 SL.; ^b^ Percentages of CD79a^+^ positive cells obtained by two-color flow cytometric panel.; ^c^ Absolute values of CD79a^+^ or CD21^+^ subset estimated by multiplying flow cytometric relative percentages and lymphocyte absolute values obtained by Cell Dyn 3700 SL.; ^d^ Percentages of CD3^+^/γδ^+^ obtained by four-color flow cytometric panel.; ^e^ Percentages of CD3^+^/γδ^−^ CD4 or CD8 positive obtained by four-color flow cytometric panel.; */** Indicates the significant difference between the vaccinated water buffalo and unvaccinated (controls) water buffaloes calculated with the * Kruskal—Wallis test (non-parametric test) or ** Student *t*-test.; ^f^ Standard error of the mean (SE).

**Table 5:** Comparison of B and T lymphocyte subset percentages between water buffalo using an inactivated gE-deleted marker vaccine (Group A) and unvaccinated control buffaloes (Group B) after challenge infected with virulent BoHV-1.

Item	Time Point (PCD)	Group A		Group B		*p*-Value *^,^**
		Mean	SE ^a^	Mean	SE ^a^	
B Lymphocytes	0	26.6	3.26	22.4	1.83	0.2945 **
	2	26.6	3.17	23.2	1.24	0.3474 **
	4	19.5	5.5	21.0	2.00	1 *
	7	26.4	2.84	20.4	1.43	0.0960 **
	10	29.0	2.95	25.6	1.72	0.3486 **
	15	25.0	3.67	23.4	1.57	0.6993 **
	30	25.4	3.71	28.8	1.56	0.4228 **
	59	33.4	3.41	28.2	2.51	0.2552 **
B Lymphocytes CD21^+^	0	80.2	3.06	78.2	1.39	0.5680 **
	2	82.8	3.51	76.2	2.71	0.0749 *
	4	85.00	3.00	80.00	2.00	0.2207 *
	7	86.2	2.08	79.8	0.37	0.1138 *
	10	86.4	1.96	80.2	0.2	0.0069 *
	15	85.8	1.66	76.6	1.69	0.0088 *
	30	87.4	2.62	73.2	2.27	0.0034 **
	59	77.4	3.06	71.6	4.58	0.3230 **
γδ T Lymphocytes	0	53.82	1.69	41.76	3.52	0.0150 **
	2	63.92	2.99	55.88	3.85	0.1378 **
	4	54.14	2.40	49.57	2.79	0.2490 **
	7	56.15	3.02	43.43	2.95	0.0167 **
	10	53.92	4.58	43.97	4.61	0.1646 **
	15	66.36	4.68	53.02	6.09	0.1206 **
	30	63.08	3.1	51.24	6.52	0.1396 **
	59	58.33	3.75	48.46	3.18	0.0795 **
αβ T CD4^+^	0	72.95	1.89	69.58	1.67	0.0758 *
	2	74.21	2.09	67.79	2.40	0.0785 **
	4	74.41	1.47	73.28	1.70	0.6283 **
	7	72.34	1.53	67.81	2.06	0.1155 **
	10	72.59	1.38	66.01	1.41	0.0103 **
	15	73.1	2.25	67.06	1.48	0.0553 **
	30	73.73	1.97	71.08	1.26	0.2884 **
	59	70.96	2.15	65.89	0.90	0.0613 **
αβ T CD8^+^	0	16.11	1.72	20.47	1.83	0.1207 **
	2	14.63	1.78	22.33	2.69	0.0440 **
	4	14.97	1.24	16.79	1.57	0.4647 *
	7	16.64	1.40	22.67	1.97	0.0377 **
	10	16.59	1.53	24.08	1.50	0.0283 *
	15	15.89	1.92	23.40	1.69	0.0190 **
	30	15.85	2.02	19.53	1.69	0.2007 **
	59	17.58	0.88	23.69	1.34	0.0294 **
αβ T CD4^+^/CD8^+^	0	4.78	0.61	3.52	0.35	0.1111 **
	2	5.50	0.90	3.25	0.45	0.0570 **
	4	5.14	0.53	4.52	0.42	0.3837 **
	7	4.53	0.54	3.1	0.31	0.0522 **
	10	4.54	0.46	2.80	0.23	0.0163 *
	15	4.94	0.71	2.94	0.25	0.0294 **
	30	5.03	0.76	3.78	0.42	0.1897 **
	59	4.28	0.57	2.82	0.18	0.0407 **

*/** Indicates the significant differences between vaccinated and unvaccinated (controls) water buffaloes calculated with the * Kruskal–Wallis test (non-parametric test) or ** Student *t*-test; ^a^ Standard Error of the mean (SE).
